# Emotional and cognitive responses to romantic breakups in adolescents and young adults: the role of rumination and coping mechanisms in life impact

**DOI:** 10.3389/fpsyt.2025.1525913

**Published:** 2025-03-28

**Authors:** Stefania Mancone, Giovanna Celia, Fernando Bellizzi, Alessandra Zanon, Pierluigi Diotaiuti

**Affiliations:** ^1^ Department of Human Sciences, Society and Health, University of Cassino and Southern Lazio, Cassino, Italy; ^2^ Department of Psychology and Health Sciences, Pegaso University, Naples, Italy

**Keywords:** romantic breakup, young adults, rumination, coping strategies, emotional well-being, avoidance coping, resilience, Italian adolescents

## Abstract

**Background:**

Romantic breakups can significantly impact the psychological well-being of young adults, affecting emotional, physical, and social domains. This study examines the roles of rumination and coping strategies in shaping adjustment to breakup-related distress, specifically focusing on Italian adolescents and young adults.

**Methods:**

A sample of 560 participants aged 17 to 22 who had recently experienced a romantic breakup completed questionnaires assessing rumination, coping strategies, and perceived impacts on life domains such as academic performance, family relationships, physical health, and emotional well-being. Correlation, regression, and mediation analyses were conducted to explore the relationships between rumination, coping strategies, and adjustment outcomes.

**Results:**

Rumination emerged as a significant predictor of negative outcomes in academic performance and physical health. Avoidance coping mediated the relationship between rumination and emotional well-being, suggesting that individuals who ruminate are more likely to adopt avoidance strategies, leading to greater emotional distress. Conversely, adaptive strategies like Positive Attitude and Problem Solving were associated with better adjustment, predicting improved academic performance and healthier family relationships.

**Conclusions:**

The findings underscore the importance of addressing rumination and avoidance coping in interventions aimed at supporting young adults post-breakup. Encouraging adaptive coping strategies, such as Positive Attitude and Problem Solving, could enhance resilience and mitigate the negative effects of relationship dissolution. This study contributes to a better understanding of coping processes in a culturally specific context, highlighting potential avenues for fostering resilience in youth facing emotional challenges.

## Introduction

The dissolution of romantic relationships, particularly during late adolescence and early adulthood, has profound psychological implications that influence emotional well-being, cognitive processing, and social adaptation. This period is characterized by intense identity exploration, emotional regulation development, and increasing autonomy from parental influences (1–3). Unlike younger adolescents, who may rely heavily on peer relationships and exhibit heightened distress due to less mature coping mechanisms (4), young adults are in a transitional phase where romantic experiences become central to self-concept and future relational expectations (5). However, young adults also possess greater cognitive resources for adaptive coping strategies, allowing for potential growth and resilience following romantic dissolution (6). Given this duality, understanding how rumination and avoidance coping interact in young adults is crucial for developing targeted interventions that address their specific emotional regulation needs.

Despite extensive research on adolescent breakups, fewer studies have examined how coping mechanisms evolve as individuals transition from adolescence into young adulthood. This study fills this gap by investigating the role of rumination and avoidance coping in a sample of young adults (17-22 years), highlighting how this developmental period shapes post-breakup adjustment.

Romantic breakups are significant stressors that disrupt emotional well-being, cognitive processing, and social functioning. Research indicates that the way individuals regulate their emotions post-breakup plays a critical role in psychological recovery. Coping strategies, defined as the cognitive and behavioral efforts used to manage emotional stress (7), can be broadly categorized into adaptive (e.g., problem-solving, social support) and maladaptive (e.g., avoidance, disengagement) approaches. Emotion regulation, as conceptualized by Gross (8), involves the modulation of emotional experiences to influence their intensity, duration, and expression. While some coping strategies, such as positive reappraisal, can promote well-being, others, like avoidance, can prolong emotional distress.

A key factor influencing post-breakup adjustment is rumination, which involves repetitive and passive focus on distressing thoughts and emotions related to the breakup (9). Although rumination is often classified as an emotion regulation strategy, it is generally maladaptive, as it fails to resolve distress and can reinforce negative thought cycles. Importantly, research suggests that individuals who ruminate excessively are more likely to engage in avoidance coping (10), a pattern that can further hinder emotional adjustment. According to the cognitive-avoidance model, excessive rumination may lead individuals to rely on avoidance as a temporary escape from distressing emotions, delaying emotional processing and exacerbating negative psychological outcomes.

Despite the growing body of research on post-breakup adjustment, there remains a need to better understand how specific cognitive and behavioral coping mechanisms influence emotional well-being following romantic dissolution. This study aims to address this gap by examining the interplay between rumination, coping strategies, and emotional outcomes in young Italian adults.

Breakups during this stage are frequently associated with psychological distress, including heightened sadness, anxiety, and diminished self-worth (11–15). Research indicates that young adults who experience relationship dissolution often engage in rumination and self-doubt, which can prolong negative emotional states and increase vulnerability to depression (12, 16–19). Among these, rumination, defined as the repetitive and passive focus on negative emotions related to the breakup, has been strongly associated with prolonged emotional distress and impaired psychological adjustment (9).

Coping strategies also play a crucial role in shaping post-breakup recovery. These strategies can be broadly categorized into adaptive (e.g., problem-solving, positive reframing) and maladaptive (e.g., avoidance, emotional disengagement) responses (20). While problem-focused coping is linked to positive adjustment, avoidance strategies, such as withdrawal, denial, or distraction, may prolong emotional distress by preventing effective emotional processing (21).

Despite extensive research on the emotional consequences of breakups, less attention has been paid to the interplay between rumination and specific coping mechanisms, particularly avoidance, in shaping emotional well-being. Existing studies have largely focused on Western, individualistic contexts, whereas the influence of collectivist cultural dynamics, such as those in Italy, remains underexplored.

This study addresses these gaps by examining the role of coping strategies in moderating the effects of rumination on post-breakup adjustment. Specifically, we explore how avoidance mediates the relationship between rumination and emotional distress, offering insights into the mechanisms underlying post-breakup recovery. By focusing on an Italian adolescent and young adult sample, this study contributes a culturally specific perspective to the literature on coping and romantic dissolution.

Given the strong familial and social ties characteristic of Italian culture, breakup distress may be amplified by social expectations and familial involvement (4, 16).

Individual differences play a critical role in determining the emotional aftermath of a breakup. Attachment theory suggests that individuals with insecure attachment styles experience more intense grief and maladaptive coping behaviors, whereas those with higher self-esteem and secure attachment demonstrate greater emotional resilience and personal growth post-breakup (2, 6, 16, 22). A key factor in post-breakup distress is the extent to which self-worth is contingent on the relationship, individuals who derive their self-esteem from romantic involvement tend to experience greater emotional turmoil upon relationship dissolution (16, 23).

Cognitive mechanisms also shape how individuals process and recover from breakups. Maladaptive cognitive responses, such as obsessive thoughts about an ex-partner or persistent attempts at reconciliation, can significantly hinder emotional adjustment (1, 24). Studies suggest that rumination, or repetitive negative thinking about the relationship, is linked to delayed emotional recovery and prolonged psychological distress (12, 25). In contemporary digital contexts, social media platforms have become a crucial factor in the post-breakup experience, offering both opportunities for connection and sources of distress. While social media allows individuals to maintain broader social ties, it also facilitates continued surveillance of an ex-partner, reinforcing ruminative thinking patterns and emotional distress (26–28). Thus, this study does not focus solely on social media effects but rather investigates broader emotional and cognitive processes, including rumination and coping strategies. Research has shown that prolonged exposure to an ex-partner’s online presence may exacerbate sadness, fuel comparisons, and impede emotional detachment, ultimately delaying psychological recovery (29).

The developmental stage of adolescence introduces additional complexities to post-breakup emotional adjustment. Adolescents, who are still forming their coping strategies and interpersonal identities, are particularly vulnerable to relationship loss, as romantic experiences play a significant role in social belonging and emotional security (4, 30). Relationship dissolution during adolescence may lead to heightened loneliness and social isolation, both of which are associated with increased mental health risks (31, 32). Social support plays a crucial role in buffering against the emotional distress associated with romantic breakups. However, the perceived quality of support, rather than its mere availability, determines whether individuals benefit from it. Higher perceived support quality is expected to enhance adaptive coping strategies (e.g., problem-solving, positive attitude), whereas lower perceived support may contribute to maladaptive responses (e.g., avoidance, rumination). The availability of social and familial support systems influences how adolescents navigate post-breakup distress. Supportive family environments and strong peer relationships serve as protective factors that help mitigate the negative psychological effects of breakups, while emotionally distant or conflict-ridden family dynamics may contribute to worsened emotional outcomes (4).

Cultural factors further shape coping mechanisms and emotional responses to romantic breakups. In the Italian cultural context, close-knit family bonds provide strong emotional support, yet they may also intensify feelings of loss and rumination due to familial expectations and social norms. Research indicates that in collectivist-leaning cultures, where interdependence and family involvement play a central role in personal decision-making, individuals may experience greater internalized pressure following a breakup, contributing to increased emotional distress and self-doubt (4, 16). This dynamic can exacerbate rumination, particularly when young adults feel their breakup is negatively perceived by family members or when coping mechanisms are not yet fully developed (12, 25). Understanding these dynamics is essential for developing effective coping strategies and interventions to assist individuals in navigating the emotional challenges associated with breakups.

Despite extensive research on the emotional consequences of breakups, fewer studies have examined how specific coping mechanisms influence the relationship between rumination and post-breakup adjustment. This study addresses this gap by investigating whether coping strategies serve as mediators in this relationship, with a specific focus on the role of avoidance coping in exacerbating emotional distress and adaptive strategies in facilitating emotional recovery. By exploring these pathways, this study contributes to a better understanding of how cognitive and behavioral responses shape emotional well-being following romantic dissolution.

### Objectives of the study

This study aims to deepen the understanding of how young Italian adults adjust to romantic breakups by examining the interplay between cognitive and behavioral responses. Specifically, the objectives are: (1) To assess the relationship between rumination and perceived impacts of a romantic breakup across key life domains, including academic performance, family relationships, physical health, and emotional well-being. By analyzing these associations, the study seeks to clarify the extent to which ruminative thought patterns contribute to prolonged emotional and physical distress post-breakup. (2) To examine how coping strategies, both adaptive and maladaptive, mediate the relationship between rumination and post-breakup adjustment, assessing their influence on emotional distress. The study focuses on how strategies like Positive Attitude, Problem Solving, and Avoidance shape individuals’ experiences following relationship dissolution, offering insights into which strategies may alleviate or exacerbate emotional difficulties. (3) To examine the role of perceived support quality in shaping young adults’ post-breakup adjustment, testing its association with both positive and negative coping mechanisms. (4) To evaluate the mediating role of Avoidance Strategies in the link between rumination and mental health outcomes. By testing this mediation, the study aims to understand whether avoidance serves as a coping mechanism that intensifies the negative impact of rumination on mental health, thus identifying potential areas for therapeutic intervention.

By exploring these objectives, this research seeks to provide insight into how young individuals process romantic losses and which factors might mitigate or exacerbate the adjustment period.

## Methods

### Participants

The study sample consisted of 560 Italian adolescents and young adults, aged between 17 and 22 years (M = 18.71, SD = .87), who had experienced a romantic breakup within the past two years. Participants reported their educational background, relationship history, and details of the breakup, including the duration of the relationship, time elapsed since the breakup, and whether they or their partner initiated the separation. These demographic characteristics were collected to provide context and enable stratification of responses. The sample was composed of 258 males (46%) and 302 females (54%), recruited through online and social media platforms targeting young adults. The inclusion criterion was having experienced the dissolution of a romantic relationship, regardless of the duration of the relationship or the time elapsed since the breakup. *A priori* power analysis was conducted using GPower (33) to ensure the study had adequate statistical power for detecting effects in multiple regression and mediation analyses. Details of the power analysis are provided in the Data Analysis section.

### Procedure

Data collection was conducted over a period of four months (between March 2022 and June 2022), leveraging both online platforms and in-person recruitment to ensure a diverse and representative sample. Participants were recruited from various Italian high schools, universities, and community centers, as well as through social media channels. To participate, individuals had to meet the following criteria: aged between 17 and 22, having experienced a romantic breakup within the past two years, and providing informed consent. Upon agreeing to participate, each individual was directed to an online survey hosted on a secure platform. This platform (Questbase) was chosen for its compliance with data protection regulations, ensuring confidentiality and anonymity. Participants first read a comprehensive information sheet outlining the purpose of the study, confidentiality measures, and their right to withdraw at any time without consequence. Consent was obtained electronically, and participants confirmed their understanding and acceptance of the terms before proceeding. The Institutional Review Board of the University of Cassino and Southern Lazio approved all study protocols to ensure adherence to ethical research standards (IRB_SUSS 23: 09-02-22). The survey was designed to take approximately 20-30 minutes to complete. To reduce potential response biases, participants were assured of the anonymity of their responses and encouraged to answer as honestly as possible. After survey completion, participants had the option to provide their contact information if they wished to receive a summary of the study’s findings; however, this information was stored separately from survey data to maintain anonymity. For data quality, the survey platform incorporated features such as automatic data saving, allowing participants to complete the survey in multiple sessions if needed. Responses were screened for completeness, and only fully completed surveys were included in the final analysis.

### Tools

#### Breakup context and circumstances questionnaire

It was designed to assess the perceived impact of a romantic breakup across four key domains: academic performance, family relations, physical health, and emotional well-being. Participants rated the extent to which their breakup affected each domain using a Likert-type scale ranging from 1 (no impact) to 5 (severe impact). Example items from this questionnaire include: “How did the breakup affect your ability to concentrate on academic tasks?” and “To what extent did the breakup impact your relationships with family members?”. The indices for the four domains were created based on theoretical conceptualization rather than exploratory factor analysis. Each domain was treated as a distinct subscale, and internal consistency was assessed. The BCCQ yielded a total Cronbach’s alpha of 0.84, with subscale reliabilities as follows: Academic Performance Impact: α = 0.79; Family Relations Impact: α = 0.76; Physical Health Impact: α = 0.81; Emotional Well-being Impact: α = 0.85. Inter-correlations among these domains were moderate (ranging from 0.30 to 0.55), supporting their related yet distinct nature. The BCCQ primarily captures perceived negative consequences of the breakup rather than resilience, with higher scores indicating greater disruption.

#### Perceived support quality

It was assessed using a 4-item scale adapted from Feeney and Collins (34), measuring participants’ satisfaction with the emotional and instrumental support received from friends, family, and peers following the breakup. Responses were recorded on a 5-point Likert scale (1 = Very Low, 5 = Very High), with higher scores indicating a greater perception of support availability and effectiveness. The scale demonstrated high internal consistency (α = 0.87).

#### Coping orientation to problems experienced - new Italian version

To assess the coping strategies used by participants after the breakup, we administered the COPE-NVI, an Italian adaptation of the original COPE questionnaire developed by Carver et al. (20) and validated for the Italian context by Sica et al. (35). The COPE-NVI includes five dimensions: Social Support (measures the tendency to seek emotional and instrumental support from others. Example item: “I seek moral support from friends and relatives”), Avoidance Strategies (assesses behaviors aimed at evading the stressor or associated emotions. Example item: “I admit to myself that I can’t deal with it, and quit trying”), Positive Attitude (evaluates the inclination to adopt a positive outlook and reinterpret stressful events constructively. Example item: “I try to learn something good from experience”), Problem Solving (measures active efforts to address and resolve the problem causing stress. Example item: “I focus on dealing with this problem, and if necessary, I put other things aside”), and Turning to Religion (assesses the tendency to seek comfort and guidance through religious or spiritual means. Example item: “I try to find comfort in my religion”). Responses are rated on a 4-point Likert scale (1 = “not at all”, 4 = “very often”). This tool demonstrated within our present sample good internal consistency with Cronbach’s alpha values. The reliability values for the COPE-NVI dimensions in our study were as follows: Avoidance Strategies: α = 0.71; Positive Attitude: α = 0.74; Problem Solving: α = 0.79; Social Support: α = 0.91; Turning to Religion: α = 0.75.

#### Ruminative response scale

To measure participants’ levels of rumination, we administered the Ruminative Response Scale (36, 37). The RRS is a 22-item questionnaire assessing individuals’ tendencies to engage in ruminative thought patterns, particularly during periods of emotional distress. Responses are rated on a 4-point Likert scale (1 = “never”, 4 = “always”), with items exploring self-focused rumination (e.g., “You write down what you are thinking and analyze it”) and negative mood-related ruminative responses (e.g., “You think about how sad you feel”). The RRS demonstrated high reliability within this sample (Cronbach’s alpha = .95).

### Data analysis

Data were analyzed using SPSS (v. 27) with an alpha level set at.05. Mediation analysis was conducted using the PROCESS macro for SPSS (38, Model 4), which allows for bootstrapped confidence intervals to estimate indirect effects. An *a priori* power analysis was performed using GPower (33) to determine the required sample size for detecting significant effects in regression and mediation models. Assuming a medium effect size (f² = 0.15), an alpha level of 0.05, and a desired power of 0.80, the required sample size was approximately 85 participants. With an actual sample size of 560 participants, the study was well-powered to detect even smaller effect sizes (f² as low as 0.02), enhancing the reliability of findings across both regression and mediation analyses. Initial descriptive statistics and correlation analyses were conducted to examine the relationships among rumination, coping strategies, and perceived impacts of the breakup across life domains (academic performance, family relations, physical and emotional well-being). Multiple regression analysis was performed to identify the predictive power of rumination and coping strategies on the perceived impact of the breakup. To assess whether coping strategies mediated the relationship between rumination and the perceived impact of the breakup, a mediation analysis was conducted using the PROCESS macro in SPSS (Model 4). The following statistical assumptions and preconditions were evaluated to ensure the validity of the multiple regression and mediation analyses. Scatterplots and partial regression plots were used to examine the linearity of relationships between the independent variables (rumination and coping strategies) and the outcome variables (perceived impacts on academic performance, family relations, health, and emotional well-being). The plots indicated approximately linear relationships, fulfilling the linearity assumption required for multiple regression. Normality of residuals was assessed through the Shapiro-Wilk test and Q-Q plots. With the larger sample size of 560, the residuals approximated a normal distribution closely, in line with the Central Limit Theorem (39). This supports the assumption of normality, even with minor deviations. The assumption of homoscedasticity was tested by examining scatterplots of residuals versus predicted values. The plots showed a relatively even spread of residuals across predicted values, suggesting that the assumption of homoscedasticity was met. Variance Inflation Factor (VIF) values were calculated to assess the independence of predictor variables. All VIF values were below the threshold of 5, indicating no multicollinearity issues and confirming that each predictor uniquely contributed to the models. The Durbin-Watson statistic was calculated to check for independence of residuals, yielding a value close to 2.0. This indicated that the residuals were uncorrelated, supporting the assumption of error independence. For the mediation analysis, the conditions outlined by Baron and Kenny (40) were checked. The initial regression confirmed a significant relationship between rumination (independent variable) and mental health impact (dependent variable). Rumination also showed a significant association with the mediator (Avoidance Strategies), and Avoidance Strategies were significantly related to mental health outcomes, validating the conditions required for conducting mediation analysis.

## Results

### Descriptive statistics

The sample consisted of 46% male and 54% female participants, with a mean age of 18.7 years (SD = 0.9), ranging from 17 to 22 years. The average duration of relationships prior to the breakup was 10.2 months (SD = 5.5), suggesting a mix of short- to mid-term relationships. On average, participants reported that their breakup occurred an average of 7.5 months prior to the study (SD = 4.2). Given the study’s focus on rumination and coping, it is important to note that prolonged emotional distress was common, with 62% of participants experiencing significant negative emotions (e.g., sadness, heartache, anger) for up to three months. Rumination, a key variable in our analysis, persisted for over six months in 12% of participants, suggesting that persistent cognitive engagement with the breakup is a frequent post-dissolution phenomenon that may influence coping strategy selection. A gender comparison indicated that women reported significantly higher levels of rumination (M = 3.5, SD = 0.7) compared to men (M = 3.0, SD = 0.6), t(558) = 4.12, p <.001. No significant gender differences emerged for avoidance strategies or social support use (p >.05).

Participants differed in their use of coping strategies, with Social Support being more frequently employed than Avoidance Strategies, F(2, 558) = 6.32, p <.01. Pairwise comparisons revealed that participants who scored higher on rumination were significantly more likely to engage in Avoidance Strategies than those with lower rumination scores, t(558) = 2.89, p <.01. These findings suggest that reliance on Avoidance may serve as a maladaptive response in individuals with higher cognitive preoccupation post-breakup. Moreover, 48% of participants responded to the breakup by seeking external distractions, 21% leaned emotionally on friends, and 11% chose to focus on sports.

Overall, 44% acknowledged that, at the time of assessment, they felt the past relationship still influenced their lives in some way, with 3% perceiving this influence as very significant. Twenty-four percent of participants believe that the past relationship negatively affects their current academic performance to a moderate degree, while 13% consider this impact to be high. For 29%, the ended relationship continues to influence their family interactions, with 6% reporting that this influence is very evident. Sixty-three percent said the breakup had significantly impacted their friendships, with 5% considering these changes very extensive. Forty-seven percent stated that the previous breakup has significantly affected their current ability to establish romantic relationships with a sense of security and peace of mind. For 15%, the previous experience heavily impacts their relational capacity. Twenty-three percent reported that the effects of the breakup also currently involve health-related aspects (e.g., body weight, sleep quality, somatization), with 9% describing these negative effects as significant. Beyond emotional and cognitive impacts, 66% of participants reported some lifestyle adjustments post-breakup, with 10% indicating substantial changes. While these shifts may reflect broader identity reevaluations following relationship dissolution, their direct influence on coping strategies remains an area for further study.

**Table 1** shows the means and standard deviations for the primary variables of interest, including rumination, coping strategies as measured by the COPE-NVI subscales, and the perceived impact of the breakup on academic performance, family relations, physical health, emotional wellbeing, and perceived support quality.

**Table 1:** Descriptive statistics for key variables.

Variable	Mean	SD	Range
Ruminative Response Scale (RRS)	3.1	0.8	1.0 - 4.0
COPE - Social Support	2.9	0.7	1.0 - 4.0
COPE - Avoidance Strategies	2.2	0.6	1.0 - 4.0
COPE - Positive Attitude	3	0.6	1.0 - 4.0
COPE - Problem Solving	2.8	0.7	1.0 - 4.0
COPE - Turning to Religion	2.5	0.75	1.0 - 4.0
Impact on Academic Performance	2.5	0.9	1.0 - 5.0
Impact on Family Relations	2.6	0.8	1.0 - 5.0
Impact on Physical Health	2.3	0.9	1.0 - 5.0
Impact on Emotional Well-being	4	0.8	1.0 - 5.0
Perceived Support Quality	3.2	0.7	1.0 - 4.0

The Ruminative Response Scale (RRS) yielded a mean score of 3.1 (SD = 0.8), indicating a moderate to high level of rumination among participants. Among coping strategies, Social Support (M = 2.9, SD = 0.7) and Positive Attitude (M = 3.0, SD = 0.6) had the highest mean scores, suggesting that participants frequently relied on these adaptive strategies. Avoidance Strategies scored lower on average (M = 2.2, SD = 0.6), reflecting less frequent use of this coping mechanism. Turning to Religion (M = 2.5, SD = 0.75) was also assessed among participants, though it was used less frequently compared to Social Support and Positive Attitude. Despite its relatively lower mean score, this coping strategy may still play a role in the emotional adjustment process for certain individuals, particularly those with strong religious beliefs. Participants rated the emotional toll of the breakup on a 5-point scale, yielding a high mean score of 4.0 (SD = 0.8). This suggests that most participants experienced the breakup as emotionally taxing, indicating significant emotional distress associated with the dissolution of their relationships. Participants also reported their satisfaction with the social support received from friends and family following the breakup on a 4-point scale, with a mean score of 3.2 (SD = 0.7). This indicates a generally positive perception of the support received, suggesting that social networks played an important role in their adjustment process post-breakup.

### Correlation analysis

**Table 2** presents the correlation matrix among key study variables, including rumination (Ruminative Response Scale, RRS), the COPE subscales (Social Support, Avoidance Strategies, Positive Attitude, Problem Solving, Turning to Religion), and the perceived impacts of the breakup on academic performance, family relationships, physical health, and emotional well-being, as well as perceived support quality.

**Table 2:** Correlation matrix of key variables.

Variables	Ruminative Response	Social Support	Avoidance Strategies	Positive Attitude	Problem Solving	Turning to Religion	Academic Performance Impact	Family Relations Impact	Physical Health Impact	Emotional Well-being Impact	Perceived Support Quality
1. Ruminative Response	—										
2. Social Support	0.45**	—									
3. Avoidance Strategies	0.60**	0.50**	—								
4. Positive Attitude	-0.30*	0.40**	-0.20	—							
5. Problem Solving	-0.25*	0.35**	-0.15	0.55**	—						
6. Turning to Religion	0.10	0.20	0.25*	0.05	0.10	—					
7. Academic Performance Impact	-0.50**	-0.30*	-0.45**	0.25*	0.20	-0.15	—				
8. Family Relations Impact	-0.40**	-0.25*	-0.35**	0.20	0.15	-0.10	0.55**	—			
9. Physical Health Impact	-0.45**	-0.35**	-0.40**	0.15	0.10	-0.05	0.50**	0.45**	—		
10. Emotional Well-being Impact	-0.55**	-0.40**	-0.50**	0.30*	0.25*	-0.20	0.60**	0.50**	0.55**	—	
11. Perceived Support Quality	-0.35**	0.60**	-0.25*	0.45**	0.40**	0.15	-0.30*	-0.25*	-0.20	-0.35**	—
12. Gender (Male = 0, Female = 1)	0.30**	0.15	0.20*	-0.10	0.12	0.20*	-0.05	0.10	0.10	0.25*	0.26*

Significant correlations are indicated with a single asterisk (*) for *p* <.05 and with two asterisks (**) for *p* <.01.

The analysis indicates that rumination is positively correlated with the impact on emotional well-being (*r* = 0.55, *p* < 0.01), physical health (*r* = 0.45, *p* < 0.01), and academic performance (*r* = -0.50, *p* < 0.01). This suggests that higher levels of rumination are associated with a more intense negative effects from the breakup in these areas. Rumination is also positively correlated with avoidance strategies (*r* = 0.60, *p* < 0.01), indicating that individuals prone to rumination are also more likely to employ avoidance strategies, potentially reflecting a cycle where rumination leads to avoidance, contributing to greater emotional difficulties.

Social support shows a significant positive correlation with positive attitude (*r* = 0.40, *p* < 0.01) and problem-solving (*r* = 0.35, *p* < 0.01), suggesting that those who seek support are also inclined to use more adaptive coping strategies. Social support is positively correlated with impact on family relations (*r* = -0.25, *p* < 0.05) and academic performance (*r* = -0.30, *p* < 0.05), indicating that individuals who seek support may experience greater resilience in these domains.

Avoidance strategies are positively correlated with impact on physical health (*r* = -0.40, *p* < 0.01) and emotional well-being (*r* = -0.50, *p* < 0.01), suggesting that avoidance coping is associated with emotional and physical difficulties post-breakup. Consistently, avoidance strategies are negatively correlated with impact on academic performance (*r* = -0.45, *p* < 0.01) and family relations (*r* = -0.35, *p* < 0.05), implying that increased reliance on avoidance may hinder adjustment in these areas.

Positive attitude shows a negative correlation with rumination (*r* = -0.30, *p* < 0.01), suggesting that a positive outlook may protect against repetitive negative thinking. Positive attitude is also positively correlated with impact on academic performance (*r* = 0.25, *p* < 0.05) and family relations (*r* = 0.20, *p* > 0.05), implying that maintaining a positive outlook may facilitate adjustment post-breakup.

Problem-solving is positively associated with social support (*r* = 0.35, *p* < 0.01) and positive attitude (*r* = 0.55, *p* < 0.01), suggesting that individuals who favor problem-solving approaches are also more likely to seek support and maintain a constructive perspective. Problem-solving is positively correlated with impact on academic performance (r = 0.20, *p* > 0.05) and family relations (*r* = 0.15, *p* > 0.05), indicating that those who engage in problem-solving may perceive fewer negative impacts in daily activities and relationships.

Turning to religion shows a positive correlation with avoidance strategies (*r* = 0.25, *p* < 0.05), suggesting that individuals who engage in religious coping may also be inclined toward avoidance behaviors. However, turning to religion does not show significant correlations with other coping strategies or impact areas, indicating that its role in adjustment post-breakup may be more complex and warrants further investigation.

Perceived support quality exhibits a strong positive correlation with social support (*r* = 0.60, *p* < 0.01), indicating that individuals who perceive higher quality support are more likely to seek and utilize social support resources. Perceived support quality is positively correlated with positive attitude (*r* = 0.45, *p* < 0.01) and problem-solving (*r* = 0.40, *p* < 0.01), suggesting that perceiving support as high-quality is associated with the adoption of adaptive coping strategies. Conversely, perceived support quality is negatively correlated with rumination (*r* = -0.35, *p* < 0.01) and avoidance strategies (*r* = -0.25, *p* < 0.05), implying that high-quality perceived support may mitigate maladaptive coping mechanisms.

Gender differences emerged in the correlation analysis, particularly in relation to rumination and coping strategies. Consistent with prior research (9), gender was significantly correlated with rumination (*r* = 0.30, *p* <.01), indicating that women reported higher levels of ruminative thinking than men. Similarly, gender showed a small but significant association with avoidance strategies (*r* = 0.20, *p* <.05), suggesting that women may be slightly more inclined to engage in avoidance-based coping post-breakup. Gender also correlated positively with turning to religion (*r* = 0.20, *p* <.05) and emotional well-being impact (*r* = 0.25, *p* <.05), indicating that women were more likely to use religious coping and report a greater emotional impact of the breakup. A notable gender difference also emerged for Perceived Support Quality (*r* = 0.26, *p* <.01), with women reporting significantly higher perceived emotional and instrumental support from their social networks than men. This finding aligns with prior research suggesting that women tend to seek and receive more social support in response to stressors (41). No significant gender differences emerged for social support, problem-solving, or positive attitude (*p* >.05), suggesting that these adaptive strategies were used similarly across genders. These findings reinforce previous literature on gendered emotional regulation patterns, particularly the greater tendency for women to engage in ruminative processing and emotional reactivity following relationship dissolution.

These findings underscore the importance of perceived support quality in influencing coping strategies and adjustment outcomes following a breakup. Interventions aimed at enhancing the quality of perceived support may promote the use of adaptive coping mechanisms, thereby facilitating better emotional and physical well-being during the adjustment process. These results suggest that rumination and avoidance strategies are associated with greater distress, with negative impacts on emotional well-being, physical health, and academic performance. In contrast, adaptive coping strategies, such as social support, positive attitude, and problem-solving, appear to improve post-breakup adjustment and promote resilience. These findings underscore the importance of encouraging the adoption of positive coping strategies and highlight the crucial role of social support in helping young people manage the emotional challenges associated with romantic separations.

### Regression analysis

A multiple regression analysis was conducted to examine the predictive power of rumination and key coping strategies (Positive Attitude, Problem Solving, Avoidance Strategies) on the perceived impact of the breakup across various life domains, including academic performance, family relations, physical health, and emotional well-being. The selection of coping strategies was based on theoretical models of adaptive and maladaptive coping (8, 20) and prior empirical findings linking these specific strategies to post-breakup adjustment (9). Other coping mechanisms, such as social support and religious coping, were initially examined but did not show significant predictive power in exploratory analyses and were thus excluded from the final regression models. Emotional Well-Being Impact was assessed as a self-reported measure of the emotional consequences of the breakup. Higher scores indicate greater subjective distress and negative emotional adjustment. This variable was used as the dependent variable in the mediation models and regression analyses.

Results showed that rumination was a significant predictor of negative impacts on academic performance (β = .45, p <.01, R² = .22, F(3, 556) = 19.87, p <.001) and physical health (β = .48, p <.01, R² = .18, F(3, 556) = 16.42, p <.001), indicating that higher levels of rumination were associated with greater reported difficulties in these areas. Similarly, avoidance coping predicted greater emotional distress (β = .41, p <.01, R² = .24, F(3, 556) = 21.63, p <.001), while positive attitude and problem-solving were linked to improved outcomes in academic performance and family relations, respectively. These findings suggest that while rumination and avoidance coping contribute to greater psychological distress, adaptive strategies such as problem-solving and positive attitude may serve as protective factors.

On the other hand, Positive Attitude was a significant predictor of better adjustment in academic performance (β = .33, p <.05), and Problem Solving was significantly associated with better outcomes in family relations (β = .29, p <.05). These findings suggest that more adaptive coping strategies, such as maintaining a positive attitude and using problem-solving techniques, contribute to more favorable outcomes in specific areas affected by the breakup. Below is the **Table 3** summarizing these regression results. These findings underscore the role of adaptive coping strategies in mitigating the negative effects of relationship dissolution and highlight areas where interventions might focus to improve young people’s resilience post-breakup.

**Table 3:** Multiple regression analysis predicting impact of breakup across life domains.

Predictor Variable	Academic Performance Impact	Family Relations Impact	Physical Health Impact	Emotional Well-being Impact
Rumination	β = .45**, p <.01	—	β = .48**, p <.01	—
Positive Attitude	β = -.33*, p <.05	—	—	—
Problem Solving	—	β = -.29*, p <.05	—	—
Avoidance Strategies	—	—	—	β = .41**, p <.01

* = p < 0.05 and ** = p < 0.01.

### Mediation analysis

A mediation analysis was conducted to determine whether coping strategies (specifically, Avoidance Strategies) mediated the relationship between rumination and the perceived impact of the breakup on emotional well-being. The purpose of this analysis was to understand if the tendency to ruminate led individuals to engage in avoidance coping, which, in turn, intensified emotional difficulties after the breakup. Using Avoidance Strategies as the mediator between rumination and emotional outcomes, the analysis revealed the following key results:

Direct Effect of Rumination on emotional Well-being: Rumination had a significant direct effect on emotional outcomes, indicating that higher levels of rumination were associated with more negative impact on emotional Well-being (β = .35, p <.01).

Indirect Effect through Avoidance Strategies: The indirect path, where rumination predicted Avoidance Strategies, which in turn predicted emotional impact, was also significant. The indirect effect was β = .19, p <.05, indicating a partial mediation. This suggests that while rumination has a direct and negative impact on emotional well-being, part of its effect is mediated through an increased likelihood of engaging in avoidance coping.

Total Effect: The total effect of rumination on emotional distress (considering both the direct and indirect paths) was significant (β = .54, p <.01), reinforcing that rumination is a strong predictor of emotional difficulties post-breakup, with Avoidance Strategies amplifying this relationship.

Individuals who engage in rumination are more likely to adopt avoidance coping, which compounds the negative impact on their emotional well-being. This finding highlights the importance of addressing both ruminative thinking and avoidance coping in interventions aimed at reducing distress following a breakup, as targeting these factors may mitigate their combined effect. This analysis underscores the intertwined role of cognitive and coping mechanisms in influencing post-breakup adjustment and suggests that interventions fostering healthier coping styles could lessen the emotional toll associated with relationship dissolution.

## Discussion

This study provides important insights into how rumination and coping strategies influence young adults’ adaptation to romantic breakups, a significant life transition often marked by emotional distress and personal upheaval. By focusing on an Italian sample, this research also highlights cultural factors that may shape coping responses and emotional processing in the context of relationship dissolution.

The findings underscore rumination as a prominent predictor of negative adjustment outcomes. Higher levels of rumination were linked with worse outcomes in academic performance and physical health, suggesting that persistent negative thoughts following a breakup may hinder functioning in multiple areas of life. These findings are consistent with extensive literature linking rumination to heightened anxiety, depression, and impaired cognitive functioning (9, 42). By repeatedly focusing on the causes and consequences of the breakup, individuals may find it more difficult to concentrate, meet academic demands, and maintain their physical health. This cognitive drain may exacerbate feelings of loss, perpetuating a cycle of emotional turmoil and distress (43–48). Consistent with prior research (9), our findings indicate that women exhibit higher rumination levels than men. This suggests that gender-specific interventions may be warranted, particularly in fostering cognitive restructuring strategies for female participants experiencing persistent negative thoughts post-breakup.

The study found that Avoidance Strategies partially mediated the impact of rumination on emotional well-being. This mediation suggests that rumination not only affects emotional distress directly but also increases the likelihood of engaging in avoidance behaviors, which can deepen emotional difficulties. The use of avoidance as a coping strategy has long been associated with negative mental health outcomes, as it prevents individuals from fully processing and adapting to challenging experiences (21, 49). Instead of promoting emotional recovery, avoidance may temporarily alleviate distress while ultimately intensifying unresolved emotions over time. These findings underscore the role of avoidance in prolonging post-breakup distress, particularly when combined with ruminative thought patterns.

On the other hand, Positive Attitude and Problem-Solving coping strategies were positively associated with more favorable outcomes, such as improved academic performance and healthier family relations. These results align with resilience and positive psychology frameworks, which emphasize the role of constructive coping in facilitating emotional recovery and personal growth (20, 50). A Positive Attitude may help individuals reframe the breakup as a growth opportunity, enhancing their resilience and reducing the intensity of negative emotions. Similarly, a problem-solving orientation allows individuals to confront challenges head-on, fostering healthier social interactions and improving relational outcomes within family dynamics (51–53).

The current findings extend the understanding of cognitive-behavioral models of distress by illustrating how rumination and avoidance contribute to a maladaptive feedback loop. The combination of ruminative and avoidant coping may obstruct the adaptive processing of emotional experiences, leading to sustained emotional and physical health impacts (54). This research reinforces the need for integrated theoretical models that account for the interplay between cognitive and behavioral coping mechanisms, particularly in emotionally charged contexts such as romantic breakups (55, 56).

The findings suggest that cultural dynamics, particularly the strong familial ties characteristic of Italian culture, may play a role in shaping coping strategies (57, 58). However, as this study did not directly compare different cultural contexts or control for cultural variables, further research is needed to determine the extent to which these influences are distinct from broader individual differences in coping responses (34, 59, 60). While previous studies (61) have also explored mediation models in the context of rumination and coping strategies post-breakup, our study provides new insights by examining avoidance coping as a mediator between rumination and emotional well-being in young adults. In contrast to earlier work, which primarily focused on direct associations between coping mechanisms and distress, our findings underscore the indirect effects of rumination through avoidance, highlighting the complex interactions between cognitive and behavioral responses in the aftermath of relationship dissolution.

### Practical implications for intervention

The study’s findings point to practical implications for mental health support aimed at young adults coping with relationship dissolution. Given the role of rumination in exacerbating emotional distress, interventions could benefit from integrating mindfulness-based techniques and cognitive-behavioral strategies to help individuals break the cycle of repetitive negative thinking (62–64). Techniques such as mindfulness meditation, cognitive restructuring, and acceptance-based approaches may empower individuals to address negative thoughts without becoming absorbed in them, fostering a more balanced emotional response (65).

Addressing Avoidance Strategies in therapeutic contexts could be instrumental in promoting adaptive coping. Programs that focus on emotional processing and exposure-based interventions may help individuals engage with and process their emotions rather than deflecting them. This shift away from avoidance could facilitate emotional growth and resilience, enabling individuals to confront and adapt to their feelings of loss (66, 67).

Educational institutions, particularly universities, are well-positioned to provide resources and workshops on healthy coping strategies for relationship stress, as many young adults experience breakups during this period. Initiatives such as peer support programs, group therapy, and psychoeducational workshops could provide students with the tools needed to cope effectively, fostering both academic success and emotional well-being (5, 68). Encouraging the development of adaptive strategies like Positive Attitude and Problem Solving could also prepare young adults for other life transitions, making resilience training an invaluable component of student services.

The pervasive use of social media also complicates emotional recovery post-breakup. Adolescents often engage in online surveillance of their ex-partners, a behavior that can prolong emotional distress and hinder the adjustment process (69, 70). Such activities reinforce cycles of rumination and may amplify feelings of inadequacy, further delaying recovery (26, 29). Practical interventions should address this by promoting digital detox strategies and educating adolescents on the emotional risks associated with excessive online monitoring. Digital literacy programs could help individuals navigate social media use responsibly and reduce its emotional toll post-breakup (71, 72).

### Limitations and future research directions

This study has certain limitations that warrant consideration. First, the use of a cross-sectional design limits the ability to draw conclusions about causality. Longitudinal studies would allow researchers to assess how coping strategies and mental health outcomes evolve over time, providing insight into whether certain coping patterns change or stabilize following a breakup.

While time since breakup is often associated with emotional recovery, it was not included as a predictor in the regression analyses due to its relatively uniform distribution across participants and its non-significant correlations with coping outcomes in exploratory analyses. However, future research may benefit from a longitudinal design to assess changes in emotional adaptation over time.

The sample was limited to Italian adolescents and young adults, which may restrict the generalizability of the findings to other cultural settings. Cultural expectations and norms likely shape individuals’ experiences and coping mechanisms, suggesting that these relationships might vary in different cultural contexts. Future research should aim to explore these dynamics cross-culturally to determine whether similar patterns of rumination, avoidance, and adaptive coping emerge.

Self-report data, while valuable for capturing subjective experiences, may also introduce biases such as social desirability and recall limitations. To complement self-report measures, future studies might incorporate observational or physiological assessments of coping behaviors and stress responses, which could yield a more comprehensive understanding of the coping process.

Although data on the duration of the relationship and the time elapsed since the breakup were collected and reported in the descriptive statistics, these variables were not included in the primary analysis. This represents a limitation, as both factors could significantly influence participants’ emotional responses and coping mechanisms. A further potential limitation of this study is that we did not assess whether participants had moved on to new romantic relationships or experienced additional breakups during the two-year timeframe considered. Future studies should consider incorporating these variables into the analysis to better understand their impact on post-breakup adjustment.

## Conclusion

In this study, we examined the coping strategies employed by young adults following romantic breakups and their effects on various life domains. Our findings indicate that rumination is significantly associated with negative impacts on academic performance and physical health, while avoidance strategies correlate with diminished emotional well-being. Conversely, positive attitude and problem-solving approaches are linked to improved academic performance and family relations, respectively. These results underscore the importance of promoting adaptive coping mechanisms to mitigate the adverse effects of relationship dissolution among young adults. Unlike prior studies, which primarily focus on individual-level predictors, this study suggests that broader social and cultural dynamics may also play a role in shaping post-breakup adjustment. Future research should continue to explore these factors in cross-cultural contexts.

## Data Availability

The raw data supporting the conclusions of this article will be made available by the authors, without undue reservation.
